# Molecular Basis for Short-Chain Thioester Hydrolysis by Acyl Hydrolases in *trans*-Acyltransferase Polyketide Synthases

**DOI:** 10.1021/jacsau.4c00837

**Published:** 2024-11-18

**Authors:** Christopher D. Fage, Munro Passmore, Ben P. Tatman, Helen G. Smith, Xinyun Jian, Upeksha C. Dissanayake, Mia E. Foran, G. Andrés Cisneros, Gregory L. Challis, Józef R. Lewandowski, Matthew Jenner

**Affiliations:** a Department of Chemistry, University of Warwick, Coventry CV4 7AL, U.K.; b Warwick Integrative Synthetic Biology Centre, University of Warwick, Coventry CV4 7AL, U.K.; c Institute for Integrative Biology of the Cell (I2BC), CEA, CNRS, Université Paris-Saclay, 91198 Gif-sur-Yvette, France; d Department of Biochemistry and Molecular Biology, Biomedicine Discovery Institute, Monash University, Clayton, VIC 3800, Australia; e ARC Centre of Excellence for Innovations in Peptide and Protein Science, Monash University, Clayton, VIC 3800, Australia; f Department of Physics, University of Warwick, Coventry CV4 7AL, U.K.; g Warwick Medical School, University of Warwick, Coventry CV4 7AL, U.K.; h Department of Physics, University of Texas at Dallas, Richardson, Texas 75801, United States; i Department of Chemistry and Biochemistry, University of Texas at Dallas, Richardson, Texas 75801, United States

**Keywords:** biosynthesis, natural products, polyketide synthases, protein−protein interactions, molecular simulations

## Abstract

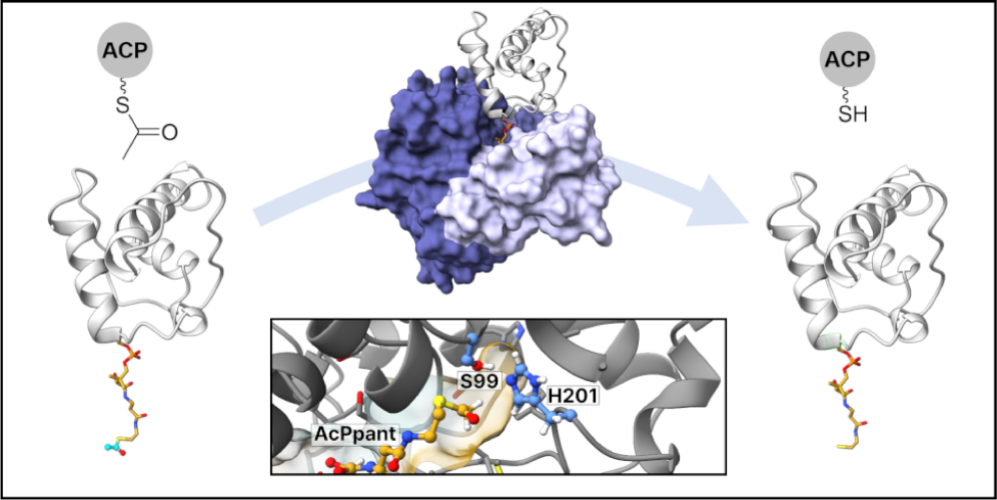

Polyketide synthases (PKSs) are multidomain enzymatic assembly lines that biosynthesize a wide selection of bioactive natural products from simple building blocks. In contrast to their *cis*-acyltransferase (AT) counterparts, *trans*-AT PKSs rely on stand-alone ATs to load extender units onto acyl carrier protein (ACP) domains embedded in the core PKS machinery. *Trans*-AT PKS gene clusters also encode stand-alone acyl hydrolases (AHs), which are predicted to share the overall fold of ATs but function like type II thioesterases (TE_II_s), hydrolyzing aberrant acyl chains from ACP domains to promote biosynthetic efficiency. How AHs specifically target short acyl chains, in particular acetyl groups, tethered as thioesters to the substrate-shuttling ACP domains, with hydrolytic rather than acyl transfer activity, has remained unclear. To answer these questions, we solved the first structure of an AH and performed structure-guided activity assays on active site variants. Our results offer key insights into chain length control and selection against coenzyme A-tethered substrates, and clarify how the interaction interface between AHs and ACP domains contributes to recognition of cognate and noncognate ACP domains. Combining our experimental findings with molecular dynamics simulations allowed for the construction of a data-driven model of an AH:ACP domain complex. Our results advance the currently incomplete understanding of polyketide biosynthesis by *trans*-AT PKSs, and provide foundations for future bioengineering efforts to offload biosynthetic intermediates or enhance product yields.

## Introduction

Type I modular polyketide synthases (PKSs) are impressive molecular machines responsible for the construction of complex, bioactive natural products that find numerous applications in both medicine and agriculture.^1,2^ Often likened to “molecular assembly lines,” these multidomain enzymes conform to a paradigm of covalent substrate attachment for exceptional processivity. Attachment occurs at the free thiol of a coenzyme A (CoA)-derived phosphopantetheine (Ppant) moiety that is post-translationally appended to *apo*-acyl carrier protein (ACP) domains by a phosphopantetheinyl transferase (PPTase) enzyme. While tethered to this thiol, acyl groups are efficiently shuttled between active sites of catalytic domains, making ACP domains central to modular PKSs. Using enzymology akin to that of fatty acid synthases, a minimal PKS chain extension module consists of an acyltransferase (AT) domain that loads an (alkyl)malonyl-derived extender unit onto the ACP domain, and a ketosynthase (KS) domain that catalyzes a Claisen-like decarboxylative condensation between the extender unit and the upstream polyketide chain to yield a β-keto-thioester intermediate. Optional α/β-carbon-modifying domains within modules, such as ketoreductase (KR), dehydratase (DH), enoylreductase (ER) and methyltransferase (MT) domains, alter the resulting β-keto-thioester to further enhance chemical diversity.^3−5^

**Figure 1:**
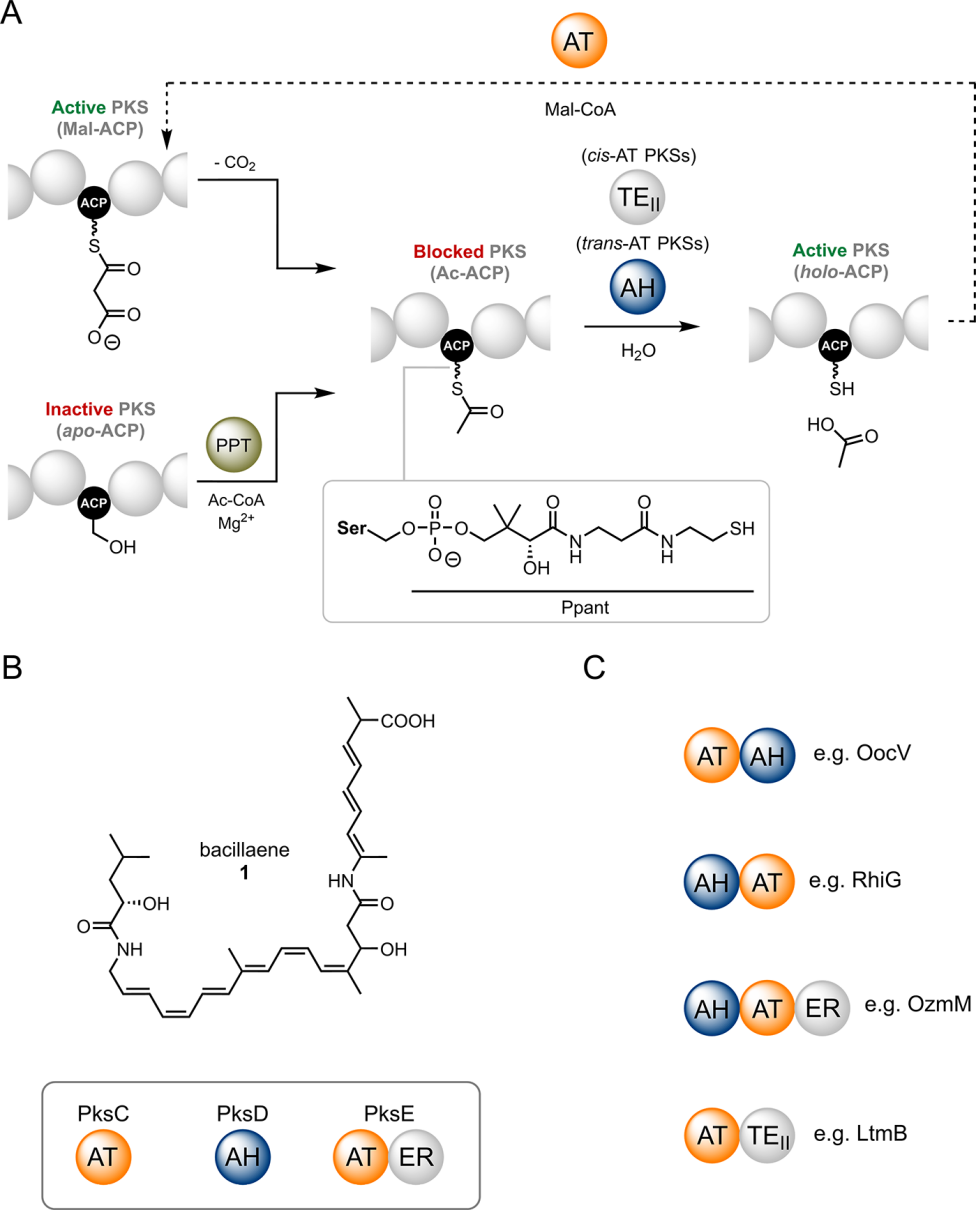
Catalytic roles of AH, AT and TE_II_ domains and their occurrence in *cis*/*trans*-AT PKSs. (A) Proofreading and extender unit selection in modular PKSs. Spontaneous decarboxylation of malonyl extender units or erroneous loading of acetyl-CoA by a PPTase (PPT; left) results in a stalled acetyl-ACP species, which blocks the PKS (center). AH and TE_II_ catalyze hydrolysis of the acetyl group (or other short, aberrant groups) to give a *holo*-ACP species (right), which can then be malonylated through AT activity (top). (B) Chemical structure of the bacillaene product of the PksX pathway, with employs the ATs and AH shown. (C) Examples of AT and AH domain fusions in *trans*-AT PKSs.

Evolution has given rise to two phylogenetically distinct classes of modular PKSs: *cis*-AT and *trans*-AT. In stark contrast to the “textbook” *cis*-AT PKSs, AT domains are absent from the chain extension modules of *trans*-AT assembly lines. Instead, the loading of extender units onto ACP domains is catalyzed by a stand-alone AT encoded by distinct genes elsewhere in the biosynthetic gene cluster (BGC).^6^ Interestingly, many *trans*-AT PKS BGCs also encode a *trans*-acting AT-like enzyme possessing hydrolytic activity toward ACP domain-bound thioesters. These enzymes, subsequently classified as acyl hydrolases (AHs), are distinct from ATs at the sequence level and form a separate phylogenetic clade.^7^ Although AHs were originally hypothesized to “proofread” PKSs by hydrolyzing any stalled intermediates from ACP domains, subsequent analysis of their substrate specificity showed a clear preference for short, linear acyl chains attached to ACP domains.^8^ This suggested a more precise housekeeping role for AHs: namely, the removal of unwanted acetyl groups from ACP domains, installed by erroneous PPTase-catalyzed transfer from acetyl-CoA or by spontaneous decarboxylation of malonyl-ACP domain thioesters (Figure 1A). Such a role is analogous to that played by type II thioesterases (TE_II_s) in *cis*-AT PKSs, which have been shown to similarly prefer short acyl chains attached to ACP domains (Figure 1A).^9,10^ Disruption of TE_II_-encoding genes in various BGCs often leads to significant losses in product yields, implying TE_II_s are crucial for biosynthetic efficiency.^11^ Thus, the coexpression of TE_II_s and/or AHs alongside PKSs normally lacking them could facilitate the formation of desired polyketide products. Despite their related functions, TE_II_s are structurally and mechanistically distinct from AHs and possess a single subdomain (instead of two) with a catalytic triad (instead of a dyad). These features highlight the disparate mechanisms that PKSs have evolved to solve key biochemical problems.

Although *trans*-acting ATs and AHs can be found as stand-alone proteins, such as PksC (AT) and PksD (AH) from the bacillaene PKS (Figure 1B), they can also occur as di- or tridomain fusions with AT-AH (e.g., OocV), AH-AT (e.g., RhiG), AT-ER (e.g., PksE) or AH-AT-ER (e.g., OzmM) domain architectures (Figure 1C).^6^ Interestingly, ATs, but not AHs, can pair with a C-terminal ER domain, possibly as a result of favorable gene fusion events. It is worth noting that the closely related lactimidomycin and migrastatin BGCs possess a *trans*-acting AT-TE_II_ didomain (LtmB/MgsB)^12^ – the only known example of a hybrid protein containing elements of both *cis*-AT and *trans*-AT PKSs (Figure 1C). Recent studies have afforded valuable insights into the structure and mechanism of *trans*-acting ATs. For example, the AT domain of DisD (AT-ER) from disorazol biosynthesis exhibits an α/β-hydrolase fold with two subdomains,^13^ similar to its *cis*-acting relatives.^14−17^ It is worth noting that AT domains from *cis*-AT PKSs are often crystallized as covalently tethered KS-AT didomains,^18,19^ while only a remnant of the KS-AT linker region (or “flanking subdomain”) can be found appended to the KS domains from *trans*-AT PKSs.^20,21^ Whereas the majority of *trans*-acting ATs use malonyl-CoA extender units, examples of alkylmalonyl-CoA extender unit utilization have been observed.^22−24^ Specificity toward malonyl extender units is achieved through conserved GH**S**xGE and xF**H**S motifs (catalytic dyad residues highlighted), where the xF**H**S motif’s Phe residue is positioned to preclude α-substituted extender units.^25^ In addition, a recently reported crystal structure in which the DisD AT domain was cross-linked to a cognate ACP domain yielded residue-level insight into the protein–protein interactions governing malonyl transfer, in close agreement with previously published alanine scanning mutagenesis results.^13,26^

Bioinformatics-based prediction of the AH structure suggests an α/β-hydrolase fold as observed for ATs; however, the mechanism by which the same overall fold catalyzes chain length-controlled hydrolysis of ACP domain-bound acyl groups has remained unclear. Herein, we report the first structure of an AH from a *trans*-AT PKS, which, when compared to AT counterparts, provides insights into mechanism and substrate specificity. Roles for key residues are further supported by intact protein-mass spectrometry (MS) assays of AH variants. In addition, the molecular basis for interactions between AHs and ACP domains is illuminated through alanine scanning mutagenesis, MS-based carbene footprinting, and molecular dynamics (MD) simulations. Finally, AH activity is demonstrated on a panel of carrier protein domains from other biosynthetic systems, highlighting the utility of AHs for dissecting machineries that depend on carrier protein domains to shuttle intermediates. Our study adds to the emerging understanding of the unique catalytic strategies employed by *trans*-AT PKSs to assemble diverse bioactive compounds and paves the way for bioengineering applications of AHs.

## Results and Discussion

### Structural Analysis of PksD

To gain insight into the differential catalytic roles of AHs and ATs in spite of a putatively shared fold, we pursued a crystal structure of a representative AH. PksD, the AH from the bacillaene pathway (Figure 1B), was a reasonable starting point because PksC, the corresponding AT from the same pathway, has been crystallized and characterized,^27,28^ providing an excellent point of comparison. The *pksD* gene from *Bacillus subtilis* str. 168 was cloned and the corresponding protein was overproduced in *Escherichia coli* as an N-terminal His_8_-tagged fusion protein, which was purified to homogeneity using immobilized metal-ion affinity chromatography and size-exclusion chromatography (Table S1). The identity of the purified protein was confirmed by ESI-Q-TOF-MS analysis (Figure S1). Crystallographic screens and subsequent optimization of both native and selenomethionine (SeMet)-derived PksD, including streak seeding for the latter,^29^ yielded diffraction-quality crystals (Figure S2A, Tables S2 and S3). A single-wavelength anomalous dispersion data set, diffracting to ∼1.96 Å from a SeMet-derived crystal, revealed four chains per asymmetric unit in a *C*2 unit cell (Table S4). One of these chains was employed as a molecular replacement search model to phase reflections from a native crystal, which diffracted to ∼2.20 Å. Eight native PksD chains were identified per asymmetric unit in a *C2* unit cell with significantly different parameters than the SeMet derivative (Table S4). Notwithstanding discrepancies in crystal packing, SeMet-derived and native PksD chains superpose very well (Figure S2B), and by virtue of the higher resolution of the anomalous data set, all structural analyses were performed on the SeMet-derived model.

**Figure 2:**
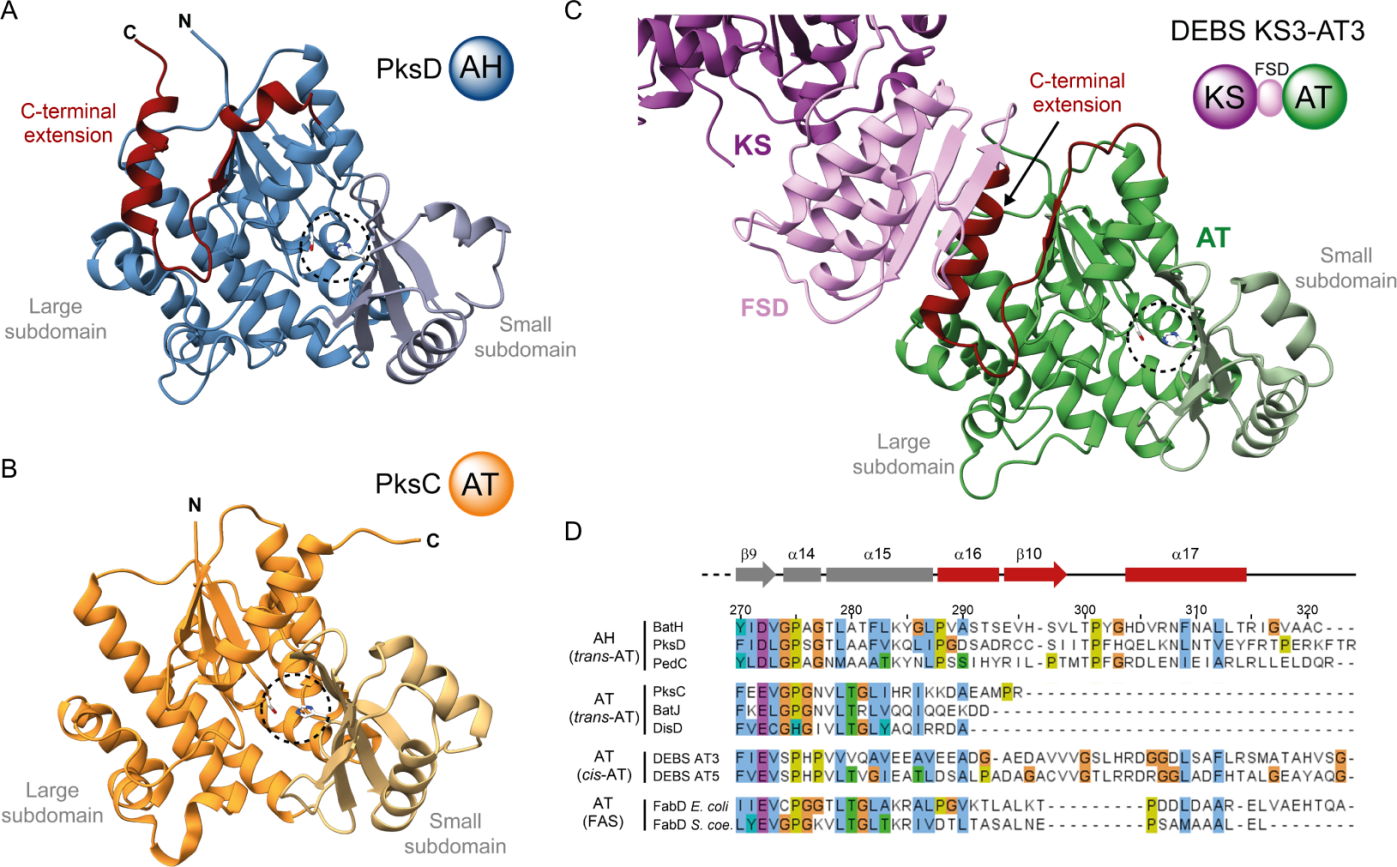
Structure of PksD and comparison to AT domains involved in polyketide biosynthesis. Catalytic residues are highlighted as sticks, and the small ferredoxin-like subdomain is colored in a lighter shade than the large subdomain. Structural comparison of (A) PksD (AH) (PDB: 8AVZ) and (B) PksC (AT) (PDB: 5DZ6) from the bacillaene *trans*-AT PKS. The overall topology of both domains is highly similar, apart from the C-terminal extension appended to PksD (highlighted in red). (C) Structure of the KS-AT didomain fragment from module 3 of 6-deoxyerythronolide B synthase (DEBS; PDB: 2QO3). In *cis*-AT PKSs like DEBS, the C-terminal extension is sandwiched between the flanking subdomain (FSD) and the large subdomain of the AT domain; a C-terminal loop wraps around the FSD and packs closely to the KS domain surface. (D) Multiple sequence alignment of AHs and ATs from *trans*-AT PKSs, *cis*-AT PKSs and bacterial type II fatty acid synthases (FASs). C-terminal sequences are shown to highlight the presence and absence of the C-terminal extension. The secondary structure elements of PksD (common among the selected domains with differing boundaries) are displayed above. Abbreviations: *S. coe.* (*Streptomyces coelicolor*). Sequence numbering relates to PksD, and accession numbers are provided in the Supporting Information.

Structural comparison of DisD (**AT**-ER) (PDB: 5ZK4),^26^ PksC (AT) (PDB: 5DZ6) and PksD (AH) (PDB: 8AVZ) reveals very similar topologies, albeit with significant deviations in the relative positions of secondary structure elements between the two *trans*-acting enzyme subtypes (compare Figure 2A,B). Conspicuously, a C-terminal extension consisting of residues Ile287-Arg324 (red in Figure 2A) is appended to helix α15 of PksD and is absent from the ATs. This feature supplies an additional 3_10_-helix, β-strand, α-helix and intervening loops that account for ∼12% of the mass of PksD (Figure S3). In *cis*-acting AT domains, such as that from the third module of the 6-deoxyerythronolide B PKS (PDB: 2QO3),^30^ the extension furnishes an interface between the flanking subdomain and the AT domain, with a C-terminal loop tracing the surface of the KS domain (Figure 2C,D). Interestingly, the C-terminal extension is present in many structures of stand-alone and embedded ATs from both primary and secondary metabolism, with variations in length (20–36 residues) arising from truncations of the unstructured loops (Figure S4).

Although PksD eluted as an apparent monomer during size-exclusion chromatography (Figure S5), we could identify a limited interface (∼1050 Å^2^ buried) between pairs of monomers in the crystal structure.^31^ Here, a copurified Zn^2+^ ion is symmetrically coordinated with tetrahedral geometry by Cys236 and His238 side chains from each of two monomers (Figure S6 and Table S5). Addition of Zn^2+^ to purified PksD at various stoichiometries resulted in precipitation, preventing further analysis of this observation. Curiously, the observed coordination pattern resembles that of zinc fingers, a family of intramolecular motifs used primarily by DNA- and RNA-binding proteins to regulate gene expression.^32^ However, a similar role is deemed improbable for AHs, especially given the lack of conservation of Cys236 and His238 (Figure S7).

### Structural and Functional Analysis of PksD Catalytic Mechanism

**Figure 3:**
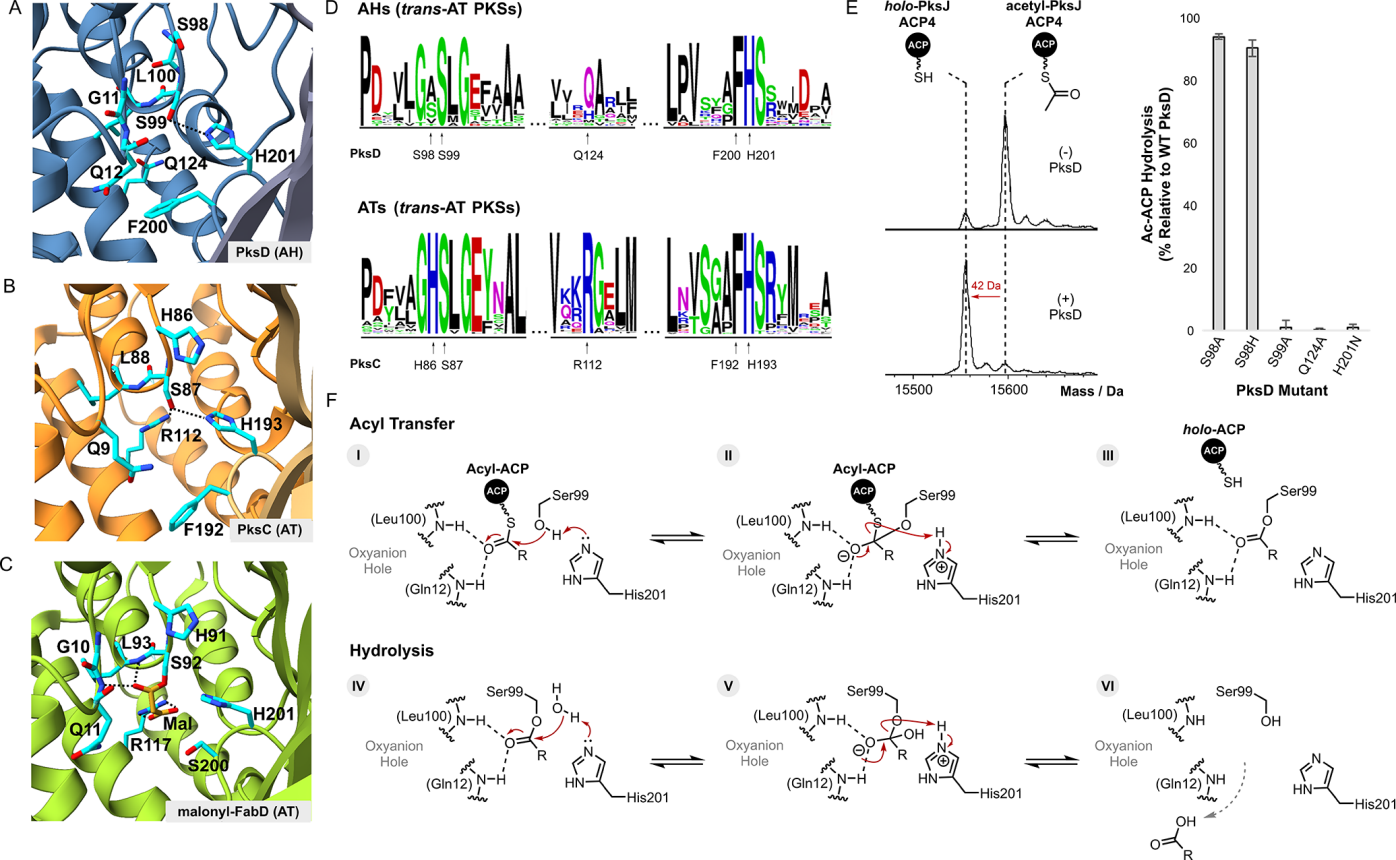
Comparison of AH and AT active sites provides insights into substrate specificity and the catalytic mechanism. Direct comparison of active site residues for (A) PksD (AH) (PDB: 8AVZ), (B) PksC (AT) (PDB 5DZ6) and (C) FabD (AT) (PDB: 2G2Z). The positively charged Arg residue in PksC and FabD is essential for acyl transfer and promotes binding of the malonyl group. Absence of an Arg residue at this position is diagnostic of an AH (usually replaced with Gln/His; see Figure S8). (D) Sequence logo comparison of active site residues in AHs and ATs from *trans*-AT PKS systems. Letter height indicates the relative degree of sequence conservation at each position. The residue type and position for PksD and PksC are shown beneath their respective sequence logos. (E) Left: stacked, deconvoluted ESI-Q-TOF spectra of the acetylated PksJ ACP4 domain before (*top*) and after (*bottom*) incubation with PksD. Right: effect of PksD active site mutations on hydrolytic activity, plotted relative to WT PksD. Error bars represent the standard deviation of three replicates. (F) Proposed catalytic mechanism for PksD-catalyzed hydrolysis of an ACP domain-tethered thioester. The R-group represents a short alkyl chain.

Akin to its AT relatives, PksD appears to employ a Ser-His catalytic dyad (Figure 3A,B,C). His201 is optimally positioned to deprotonate the hydroxyl group of Ser99 (<3 Å between Nε and Oγ), thus activating it for nucleophilic attack on incoming ACP domain-bound substrates; His201 may likewise be poised to activate a water molecule for hydrolytic cleavage following acylation of Ser99 with the substrate (see our MD simulations below). *E. coli* FabD (PDB: 2G2Z), an AT involved in fatty acid biosynthesis, was previously crystallized in a noncovalent complex with CoA and with the nucleophilic Ser92 acylated with a malonyl group.^33^ The positively charged guanidino group of the conserved Arg117 residue forms a stabilizing salt bridge with the negatively charged carboxylate group of the malonyl moiety (Figure 3C). In AHs, however, no such stabilization is possible as this position is occupied by Gln (Gln124 in PksD) or, less frequently, other residues (Figures 3A,D and S8). Substituting the conserved Arg side chain for a shorter and uncharged one is presumably important in preventing correctly loaded extender units (e.g., malonyl and methylmalonyl groups) from being transferred to and/or hydrolyzed by the AH. In line with these observations, previous work showed that mutating the malonyl-binding Arg to Gln in PedD, a *trans*-acting AT from the pederin pathway, afforded acylation of the nucleophilic Ser residue with an acetyl group carried by an ACP domain, but not subsequent hydrolysis.^8^ In another distinctive feature, the putative catalytic Ser99 residue of PksD is often preceded by another Ser residue instead of the strictly conserved His residue found in ATs (e.g., G(A/S/V)**S**L vs GH**S**L motif; catalytic Ser residue highlighted) (Figure 3D). No role has been ascribed to the residue at this position despite its strong conservation in ATs.

To evaluate the contributions of residues in the active site of PksD, we prepared several variants and compared their hydrolytic activities to those of the wild-type (WT) enzyme. The PksJ ACP4 domain, a cognate ACP domain of PksD from the bacillaene *trans*-AT PKS, was enzymatically loaded with an acetyl group, and then incubated with WT PksD or mutants thereof for 5 min, after which the reaction was acid-quenched and analyzed by intact protein-MS. In this assay, WT PksD showed near-complete hydrolytic conversion of the acetyl-PksJ ACP4 domain to the *holo*-PksJ ACP4 domain after 5 min (Figures 3E and S9). In contrast, mutation of either residue in the putative catalytic dyad (S99A and H201N) fully abolished hydrolytic cleavage of the acetyl thioester group (Figure 3E), suggesting critical roles not dissimilar from those of the equivalent pair in ATs. Given the strict conservation of His in the nucleophilic GH**S**L motif of ATs, we analyzed the corresponding Ser residue in the G(A/S/V)**S**L motif of PksD. That S98A and S98H mutations had little effect on the enzyme’s ability to hydrolyze substrate (Figure 3E) suggests a noncrucial role for the residue preceding the nucleophilic Ser in AHs, as further supported by a lack of strict conservation at this position (Figure 3D). Conversely, a Q124A mutation resulted in inactivity toward the acetyl-PksJ ACP4 domain (Figure 3E), indicating an important role for the Arg-substituting residue in either catalysis or structure (e.g., organizing the geometry of the substrate binding pocket; see below).

Taken together, these data indicate that the AH reaction likely proceeds through an acyl-enzyme intermediate similar to that formed by ATs during acyl transfer but in the reverse direction (i.e., acyl transfer from the ACP domain to the AH; top of Figure 3F). In further contrast to ATs, which partition their acyl-enzyme intermediates down acyl transfer (productive) or hydrolysis (nonproductive) pathways according to substrate viability,^34^ AHs have evolved to productively couple acyl transfer with hydrolysis (bottom of Figure 3F).

### Insights into PksD Substrate Specificity and Chain Length Control

**Figure 4:**
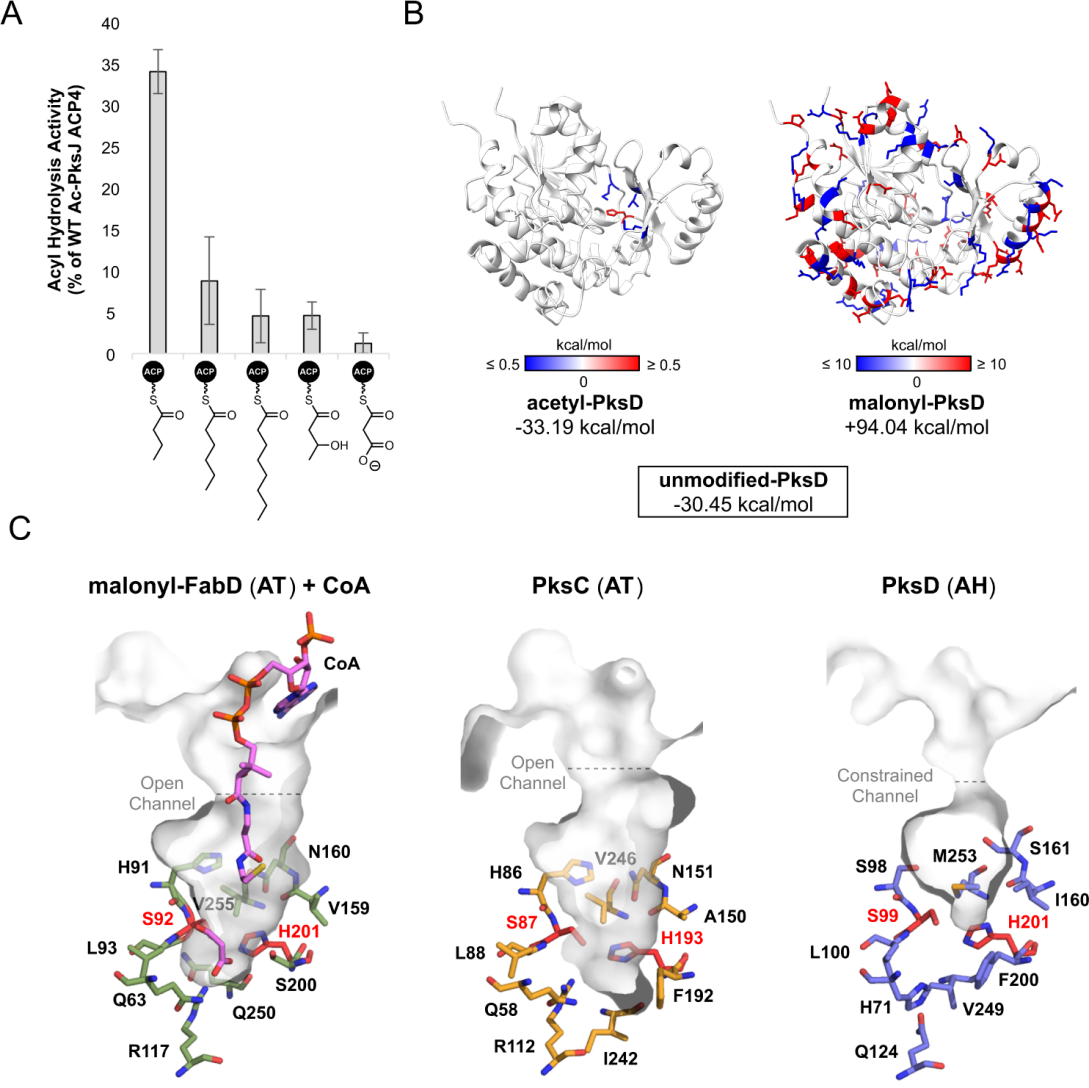
Substrate scope of PksD and analysis of the acyl chain binding pocket. (A) Bar chart showing PksD-catalyzed hydrolysis of acyl-PksJ ACP4 species (along *x*-axis) relative to the acetyl-PksJ ACP4 domain. PksD exhibits a clear preference for short, unbranched acyl chains. Only trace hydrolysis was detected for the malonyl-ACP species, in agreement with previous observations that AHs are unable to process such substrates. Error bars represent the standard deviation of three replicates. (B) Total interaction energy of Ser99-modified PksD with respect to the unmodified form. Differences in energy between acetyl-bound and unmodified (*left*) vs malonyl-bound and unmodified (*right*) are shown. A negative interaction energy stabilizes the system (blue), whereas a positive interaction energy destabilizes the system (red). Major stabilizing and destabilizing residues are highlighted on the structure of PksD for the acetylated form (>± 0.5 kcal/mol) and malonylated form (>± 10 kcal/mol). (C) Juxtaposition of the substrate binding pockets of FabD (PDB: 2G2Z), PksC (PDB: 5DZ6) and PksD (PDB: 8AVZ), suggesting a more constrained pocket and entrance channel in PksD. Pockets/channels are rendered as gray semitransparent surfaces, with select residues shown as sticks colored by atom.

Previous work showed that PedC, the AH from the pederin pathway, preferentially hydrolyses short, unbranched acyl chains bound to ACP domains.^7,8^ To determine whether this substrate profile is conserved among AHs, we measured PksD-catalyzed hydrolysis of the acetyl-, butyryl-, hexanoyl-, octanoyl-, β-hydroxybutyryl-, and malonyl-PksJ ACP4 domain using our intact protein-MS assay. Indeed, our experiments confirmed a clear preference for short, unbranched acyl chains on the PksJ ACP4 domain, with only trace hydrolysis of the malonyl group, in line with our expectations of PksD as a housekeeping enzyme (Figure 4A). In addition, computational energy decomposition analysis of acetyl- and malonyl-PksD relative to the unmodified form showed pronounced differences. Here, the total interaction energies of acetyl-PksD and the unmodified form were comparable and favorable (approximately −30 kcal/mol), whereas that of malonyl-PksD was unfavorable (approximately +94 kcal/mol), with numerous residues affected across the protein structure, indicating a suboptimal substrate (Figures 4B and S10).

Understanding how AHs exert control over substrate specificity could facilitate rational engineering efforts to broaden the substrate tolerance, allowing diverse intermediates to be off-loaded and thus *trans*-AT PKS pathways to be dissected. To study this aspect, we turned to our X-ray structure. As in ATs from PKSs, Phe200 (strictly conserved) and the oxyanion hole-forming Gln12 (strictly conserved as Gln/His) of PksD are near the nucleophilic Ser99 residue (Figure 3A,B,D). Juxtaposing a single chain of PksD (AH) with those of PksC (AT), PksE (**AT**-ER) (PDB: 5DZ7), and DisD (**AT**-ER) reveals a less accessible substrate binding pocket in the former (Figures 4C and S11–S13). In PksD, the longer and generally less structured region between helices α3 and α4 (i.e., loop α3/α4), the Phe200-containing loop β6/α10 and the Gln12-containing loop β1/α1 associate through a series of van der Waals interactions involving Gln12, Gly13, and Gln15-Tyr17 (loop β1/α1); Lys58, Val60, and Pro63-Asp65 (loop α3/α4); Ile67 (helix α4); Pro195-Tyr198 and Phe200 (loop β6/α10); Ile128 (helix α6); and Val249 (helix α12), in addition to other residues. This intimate arrangement is stabilized by an extensive H-bonding network involving Phe64 (loop α3/α4), Gln15 (loop β1/α1), Gln12 (loop β1/α1), His71 (helix α4), Gln124 (helix α6), and Phe245 (helix α12); the oxyanion hole-forming Gln12 is also H-bonded to Ser70 (helix α4) (Figure S11). Ordered water molecules further confine these three loops, mediating H-bonds between Asp65 (loop α3/α4) and Ser197 (loop β6/α10), between Tyr17 (loop β1/α1) and both Asp62 and Val60 (loop α3/α4), and between Gln15 (loop β1/α1) and Phe64.

As well as restricting access to the active site of PksD, the aforesaid interactions constrain the positions of key side chains relative to the nucleophilic Ser99 to a greater extent than in ATs (Figures S11 – S13). For example, the compact side chain of Gln124 (substituted by an Arg residue in ATs) participates in the aforementioned H-bonding network, drawing Val249 nearer to itself and to Phe200 and thereby providing hydrophobic and steric bulk that clashes with the charged malonyl group. The H-bonding network and van der Waals contacts also restrain the Gln12 side chain, which rests above the Phe200 phenyl ring and again clashes with the malonyl group. Further van der Waals interactions with Ile67, His71, Ile128, Met137, Val196, Ser197, Tyr198, His201, and Ile205 confine Phe200 in close proximity to Ser99. All of these factors likely limit the chain length and degree of branching of incoming substrates, with occasional thermal fluctuations allowing for exceptions.

That Gln124 is essential in shaping the substrate binding pocket of PksD is reflected in our finding of the abolished activity for the Q124A variant (Figure 3E). Although we sought to interrogate the role of Phe200, an F200A mutation failed to yield soluble protein, indicating that the phenyl ring–closely packed against the side chains of Met137, Ile160 and Val249–is indispensable for proper folding of the AH. While H71A and V249A mutations produced soluble protein and were expected to expand the binding pocket, both mutations abolished hydrolytic activity toward all acyl-ACP substrates. Remodelling of the PksD active site or population of the larger hydrophobic binding pocket with water molecules separated from the bulk solvent might explain these observations. Taken together, PksD clearly adopts a very subtle mechanism for achieving substrate control, resulting in an active site architecture that is sensitive to point mutations. Producing a stable and active hydrolase with an expanded substrate profile, while desirable, may require a more exhaustive engineering campaign (e.g., involving directed evolution) beyond the scope of this work.

### Analyzing PksD-Catalyzed Hydrolysis via Molecular Dynamics and Quantum Mechanics/Molecular Mechanics (QM/MM) Simulations

**Figure 5:**
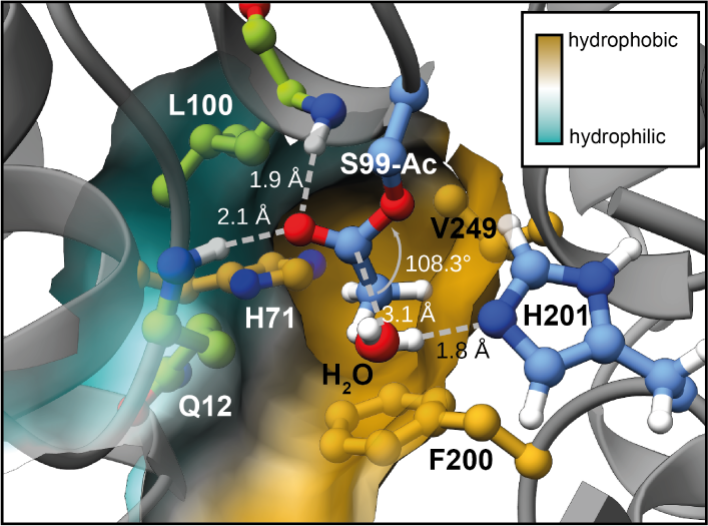
Snapshot of the catalytic geometry for hydrolysis of acetyl-PksD. Example frame from cMD simulation of PksD with an acetyl group bound to the catalytic Ser99 residue in which distance and angular requirements for nucleophilic attack by a water molecule are satisfied. The backbone NH atoms of Gln12 and Leu100 (green C atoms) form the oxyanion hole. Positioning of the acetyl group with respect to His201 and water is aided by nestling of the methyl group against the Phe200, Val249 and His71 side chains (gold C atoms). The semitransparent surface rendering is colored yellow (hydrophobic) or cyan (hydrophilic).

To more closely examine the mechanism of PksD-catalyzed acyl hydrolysis (i.e., steps IV–VI in Figure 3F), we performed a series of classical MD (cMD; 50 ns) simulations followed by accelerated MD (aMD; 320 ns) on PksD with the Ser99 residue acetylated. In numerous aMD frames, water (WAT) molecules met the distance (His201[Nε2] → WAT[H] ≤ 3 Å, WAT[O] → Ac[C1] ≤ 3.2 Å) and angular (WAT[O]-Ac[C1]-Ac[O] = 100°–110°; i.e., approaching the Bürgi-Dunitz angle) requirements for a near-attack conformation.^35^ In 10 ns cMD simulations initiated from several such frames, the His201 side chain frequently recruited water molecules (Figure S14) meeting the attack criteria. In a representative frame (Figure 5 and Video S1), the carbonyl group of the substrate is activated toward nucleophilic attack by an oxyanion hole formed by Gln12 and Leu100. The methyl group of the substrate nestles against the side chains of His71, Phe200, Val249, and Gln12, which helps position the carbonyl group. Moreover, His71 is wedged between Phe200 and Val249 and stabilized by H-bonds to the side chains of Gln12 and Gln124 (as in Figure S11A,B). In the representative snapshot (Figure 5), the methyl group of the substrate occupies a small binding pocket at the back of the active site, which cannot accommodate larger substituents. Larger substrates can only be accommodated by occupying the channel below this pocket; while the channel is mostly hydrophobic, a hydrophilic patch lining one side of it would disfavor binding of longer hydrocarbon chains (Figure S15 and Video S2).

In the case of malonyl-PksD, the geometry required for catalysis could only be met if the carboxylate group were to occupy a space similar to that of the methyl group in the acetyl substrate–an arrangement disfavored by the hydrophobic nature of the pocket. Indeed, in a 215 ns cMD simulation with malonyl-PksD, the carboxylate group moves away from the hydrophobic part of the pocket, which causes the carbonyl group to point toward His201 in a catalytically unviable conformation (Figure S16 and Video S3). Taken together, these findings explain the inverse correlation we observed between the hydrolytic efficiency of PksD and the length of the alkyl chain, as well as the inactivity of PksD toward malonyl substrates (Figure 4A).

QM/MM calculations were performed to investigate the mechanism of the hydrolysis reaction. No catalytically competent structures were found for the malonyl-bound system (all reaction energies were highly endoergic). Representative structures from the MD trajectories for acetyl-PksD were selected to obtain optimized reactant and final product structures (Figure S17, panels I and VI). An initial reaction path approximation connecting the reactant to the final product was obtained with the quadratic string method.^36^ Based on this initial approximation, and given that the complete mechanism involves global conformational changes, we performed constrained optimizations of selected structures to obtain a reaction profile associated with a possible mechanism—i.e. deprotonation of a water molecule by His201 (6.1 kcal mol^–1^; Figure S17, panel II) and concomitant nucleophilic attack of the resulting hydroxide ion at the substrate’s carbonyl group (8.4 kcal mol^–1^; Figure S17, panel III). Neutralization of Ser99 via proton transfer from His201 (23.3 and 14.3 kcal mol^–1^; Figure S17, panel IV and panel V) leads to the final product, giving a slightly endoergic energy of 0.9 kcal mol^–1^ (Video S4).

The calculated energies associated with the structures along the reaction path are consistent with energies for enzymatic catalysis.^37,38^ The relatively high energy associated with the final proton transfer step is due to the suboptimal arrangement of His201 and Ser99, which could be ameliorated by possible dynamics of the system, although no large conformational fluctuations for these residues were observed during the MD simulations. Moreover, alternative pathways could be possible, such as product release prior to the proton transfer, which could allow for larger fluctuations of the His201/Ser99 pair, or utilization of a bridging water molecule. However, these alternative processes could not be tested due to computational limitations.

### Investigating the Activity of PksD toward Pantetheine and CoA Thioesters

**Figure 6:**
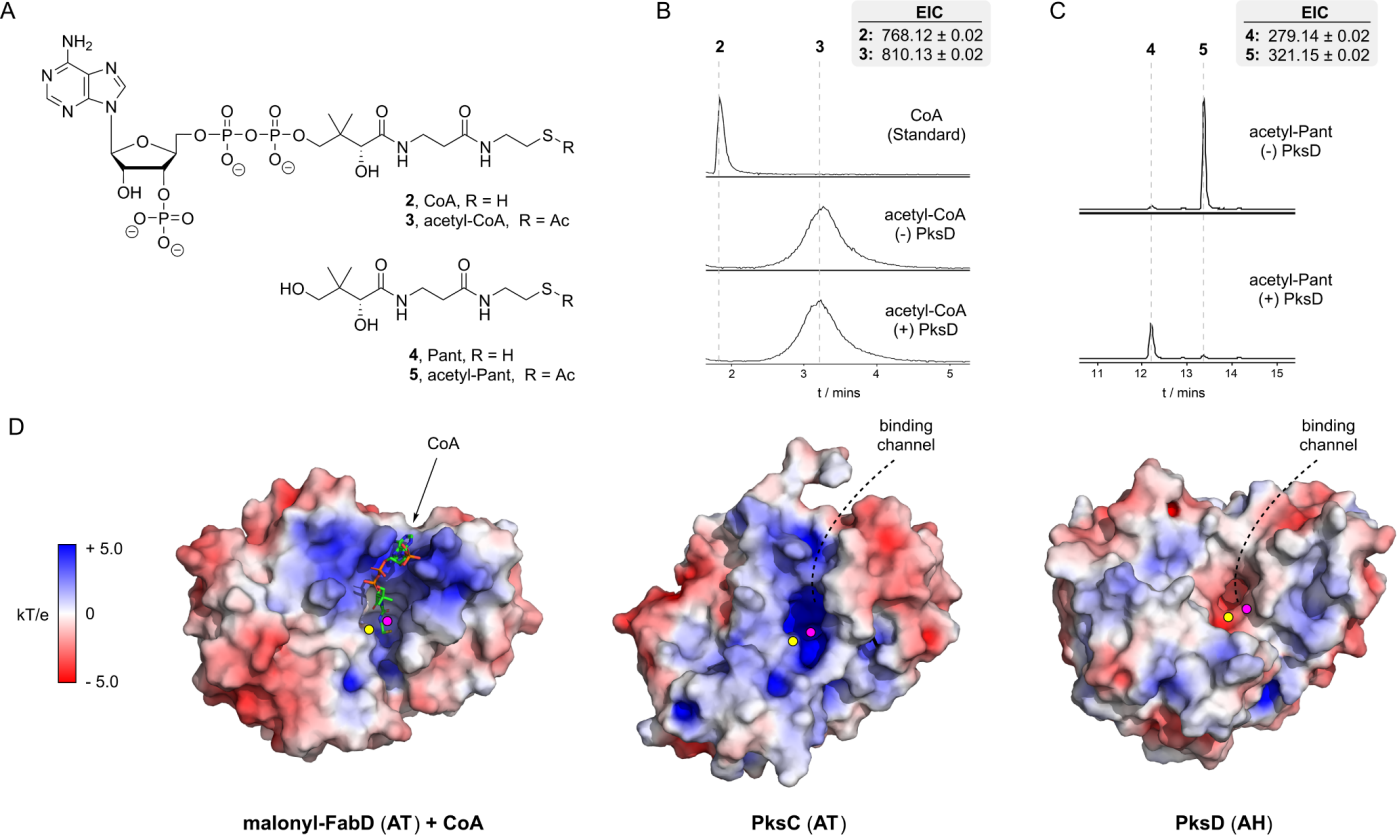
PksD selects against thioesters of coenzyme A. (A) Chemical structures of CoA, acetyl-CoA, Pant and acetyl-Pant (**2** – **5**, respectively). (B) **–** (C) Extracted ion chromatograms (EICs) from UHPLC-ESI-Q-TOF-MS analyses of PksD with acetyl-CoA or acetyl-Pant. (B). No hydrolysis of acetyl-CoA was observed in the presence or absence of PksD (bottom and middle chromatograms, respectively) by comparison to a CoA standard (top chromatogram). (C) After 3 h, complete conversion of acetyl-Pant to Pant was observed in the presence of PksD (bottom chromatogram), while only trace Pant was observed in the negative control lacking enzyme (top chromatogram). (D) Structures of FabD (PDB: 2G2Z), PksC (PDB: 5DZ6) and PksD (PDB: 8AVZ) displaying solution-phase surface electrostatic maps (calculated using APBS). The entrances to ATs carry CoA- and malonyl-stabilizing positive charges, whereas the same regions of AHs carry no or negative charges. Positions of catalytic residues are highlighted as colored circles (Ser = yellow, His = magenta).

The efficient hydrolytic activity of AHs toward short-chain acyl thioesters of ACP domains and N-acetylcysteamine^7,8^ raises questions about the fate of similar CoA thioesters in the native cellular milieu. Given their structural homology to ATs, which accept CoA thioesters for acyl transfer, AHs should process CoA thioesters, unless a gatekeeping mechanism has evolved to prevent their hydrolysis. To test for such a mechanism, we began by probing the ability of PksD to process acetyl-CoA (Figure 6A), using the same molar ratio as in assays with the acetyl-PksJ ACP4 domain. After 3 h, no PksD-mediated hydrolysis of acetyl-CoA was observed via LC-MS (Figure 6B). We next performed this reaction in the presence of acetyl-pantetheine (acetyl-Pant; Figure 6A), which lacks the 3′-phosphoadenosine moiety of CoA but closely emulates the acetylated Ppant moiety of ACP domain-bound substrates. In contrast to acetyl-CoA, acetyl-Pant was fully hydrolyzed by PksD (compared to trace hydrolysis observed in an enzyme-free control; Figure 6C).

To explain these findings, we inspected APBS-calculated electrostatic potentials along the surfaces of FabD (AT), PksC (AT) and PksD (AH).^39^ While the channels leading to the active sites of the ATs are laden with positive charges that stabilize the negatively charged CoA and malonyl moieties, the corresponding regions of PksD carry either negative or null charges that repel such groups (Figure 6D). The exclusion of malonyl groups by AHs is consistent with our experiments and those previously reported.^7,8^

### Mapping the AH-Binding Epitope of the PksJ ACP4 Domain Using Alanine Scanning Mutagenesis

Several structures of ATs and ACP domains in complex and in isolation have provided insight into their protein–protein interaction interfaces, particularly the AT:ACP domain complex from the disorazol *trans*-AT PKS.^26^ Given the high degree of structural homology between ATs and AHs, we examined whether the two enzyme types exhibit similar interaction patterns toward their cognate ACP domains.

**Figure 7:**
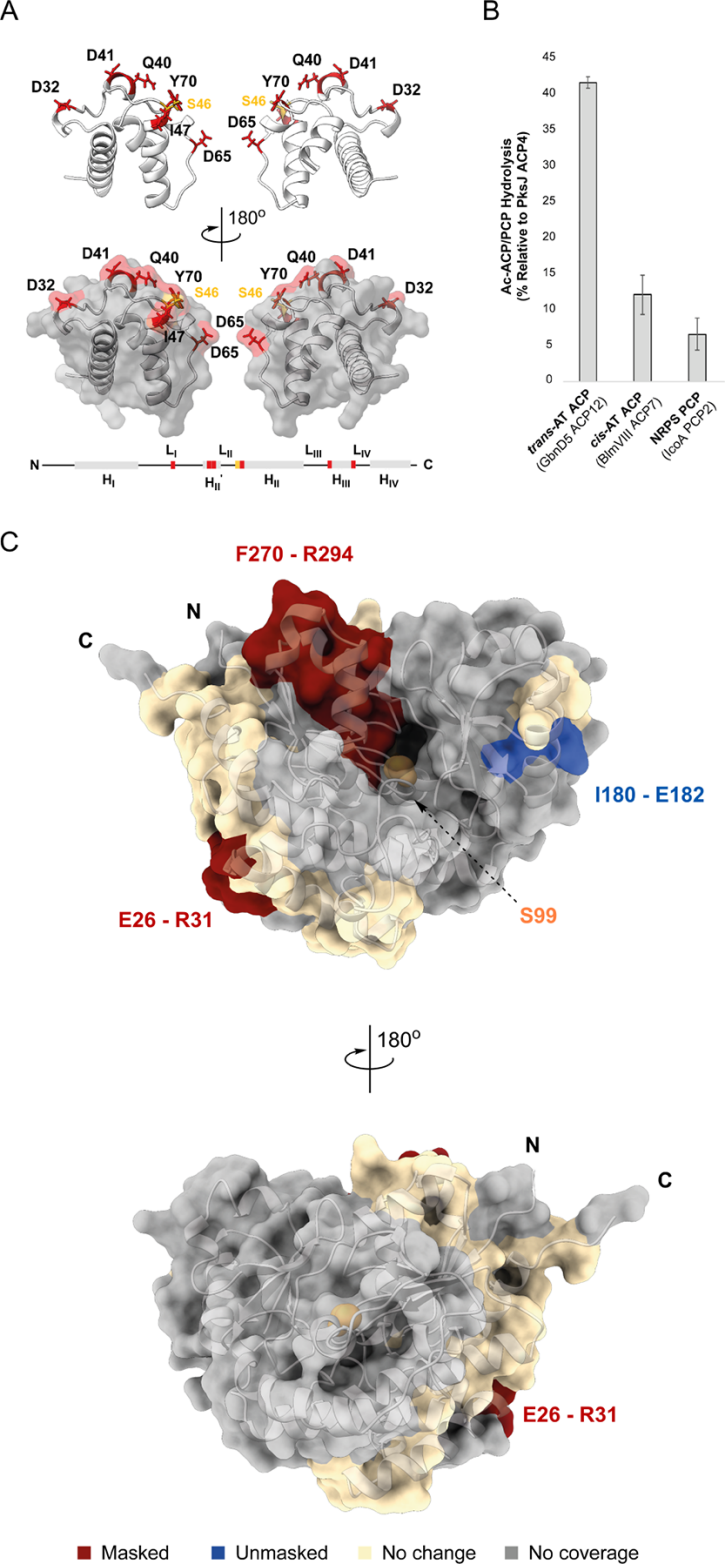
Identification of the PksD:PksJ ACP4 domain interaction epitope by scanning alanine mutagenesis and carbene footprinting. (A) AlphaFold structure of the PksJ ACP4 domain with significantly disruptive mutations (red) and nucleophilic Ser46 (orange) shown as sticks. Surface renderings of the ACP domain reveal a solvent-accessible interaction epitope. A linear representation of the PksJ ACP4 domain structure is shown. (B) Bar chart showing PksD-catalyzed hydrolysis of the acetyl group from select carrier protein domains relative to the PksJ ACP4 domain. PksD exhibits a clear preference for ACP domains from *trans*-AT PKSs, with <15% activity toward ACP/PCP domains from *cis*-AT PKS and NRPS pathways. Error bars represent the standard deviation of three replicates. (C) Structure of PksD showing the locations of masked, unmasked, and unaffected peptide regions resulting from trypsin and GluC digests in the presence of the *holo*-PksJ ACP4 domain. An extended masked region is situated at the entrance to the active site cavity, which is the likely docking site of the ACP domain. The localized unmasking is attributed to conformational changes in PksD upon ACP domain binding, as observed during MD simulations (Figure S29).

Inspired by previous successes,^13,40^ we employed an alanine scanning mutagenesis approach to define the AH:ACP domain interaction epitope on the PksJ ACP4 domain, using a previously reported library of X → Ala mutants.^41^ By testing these acetyl-loaded mutants for PksD-catalyzed hydrolysis in our intact protein-MS assay, loss of productive interaction at the AH:ACP domain interface could be pinpointed. Six residues, when individually truncated to a methyl side chain, led to a significant decrease in PksD-catalyzed hydrolysis: Asp32, Gln40, Asp41, Ile47, Asp65 and Tyr70 (Figures 7A and S18). Notably, circular dichroism spectra of these variants closely resemble that of the WT PksJ ACP4 domain, suggesting that no significant perturbation of secondary structure occurred upon mutation (Figure S19). Mapping the six residues onto an AlphaFold model of the PksJ ACP4 domain allowed a binding epitope to be visualized,^42,43^ with most of the residues proximal to the point of Ppant attachment (i.e., Ser46) (Figure 7A). The positioning of these key residues is in good agreement with the reported AT:ACP domain interfaces from *trans*-AT PKSs (e.g., DisD AT:DisA ACP1 domain and KirCII AT:KirAII ACP5 domain),^40,44^ suggesting AHs and ATs bind the “top” face of the ACP domain through a highly similar mode (Figure S20).

To expand this analysis, we examined the capacity of PksD to functionally interact with noncognate carrier protein domains, including peptidyl carrier protein (PCP) domains from functionally related nonribosomal peptide synthetase (NRPS) assembly lines. We applied our intact protein-MS assay to a selection of acetyl-loaded carrier protein domains: the GbnD5 ACP12 domain (from the gladiolin *trans*-AT PKS),^24,45^ the BlmVIII ACP7 domain (from the bleomycin *cis*-AT PKS-NRPS)^46^ and the IcoA PCP2 domain (from the icosalide NRPS)^47^ (Figure 7B). Incubation with PksD resulted in 41 ± 8%, 12 ± 1%, and 7 ± 3% acetyl group hydrolysis, respectively, relative to the acetyl-PksJ ACP4 domain. While our findings demonstrate a clear preference for acetyl-loaded carrier protein domains from *trans*-AT PKSs, those from *cis*-AT PKSs and NRPSs could also be processed, albeit at much slower rates. Variation of key residues involved in binding PksD appears to be responsible for the decreased activity against noncognate carrier protein domains, with several instances of charge reversal likely resulting in a suboptimal interaction interface (Figure S21).

### Elucidating the ACP Domain-Binding Interface of PksD by Carbene Footprinting

Subsequently, we sought to visualize the interaction epitope from the perspective of the AH. Deletions of helix α17 (Δ302–324) and helices α16-α17 (Δ290–324) in the C-terminal extension yielded insoluble protein, precluding downstream analysis but indicating this region as important for correct folding. We next turned to carbene footprinting, a structural MS technique wherein masking or unmasking of residues due to decreased or increased solvent accessibility, respectively, is detected by LC-MS analysis after covalent modification with a carbene probe and proteolytic cleavage into peptides.^48^ This technique has been employed in mapping the interactions between ACP domains and their catalytic partners in related systems.^41,45,49^

When we applied this approach to the PksD:*holo*-PksJ ACP4 domain complex, we observed a localized masked region consisting of residues 270–294 (strand β9 - helix α16) of the AH. These residues are situated at the entrance to the substrate binding pocket and comprise the equivalent surface occupied by ACP domains in reported AT:ACP domain complexes (Figures 7C and S22).^26,50^ Most other detected peptides showed no change in the presence of the ACP domain; however, parts of helix α2 (residues 26–31) and helix α9 (residues 180–182) were masked and unmasked, respectively, suggesting subtle conformational shifts in both subdomains. This is unsurprising given the previously reported conformational mobility of ATs,^51,52^ and highlights the dynamic nature of these interactions.

### Computational Docking and Analysis of the PksD:PksJ ACP4 Domain Complex

**Figure 8:**
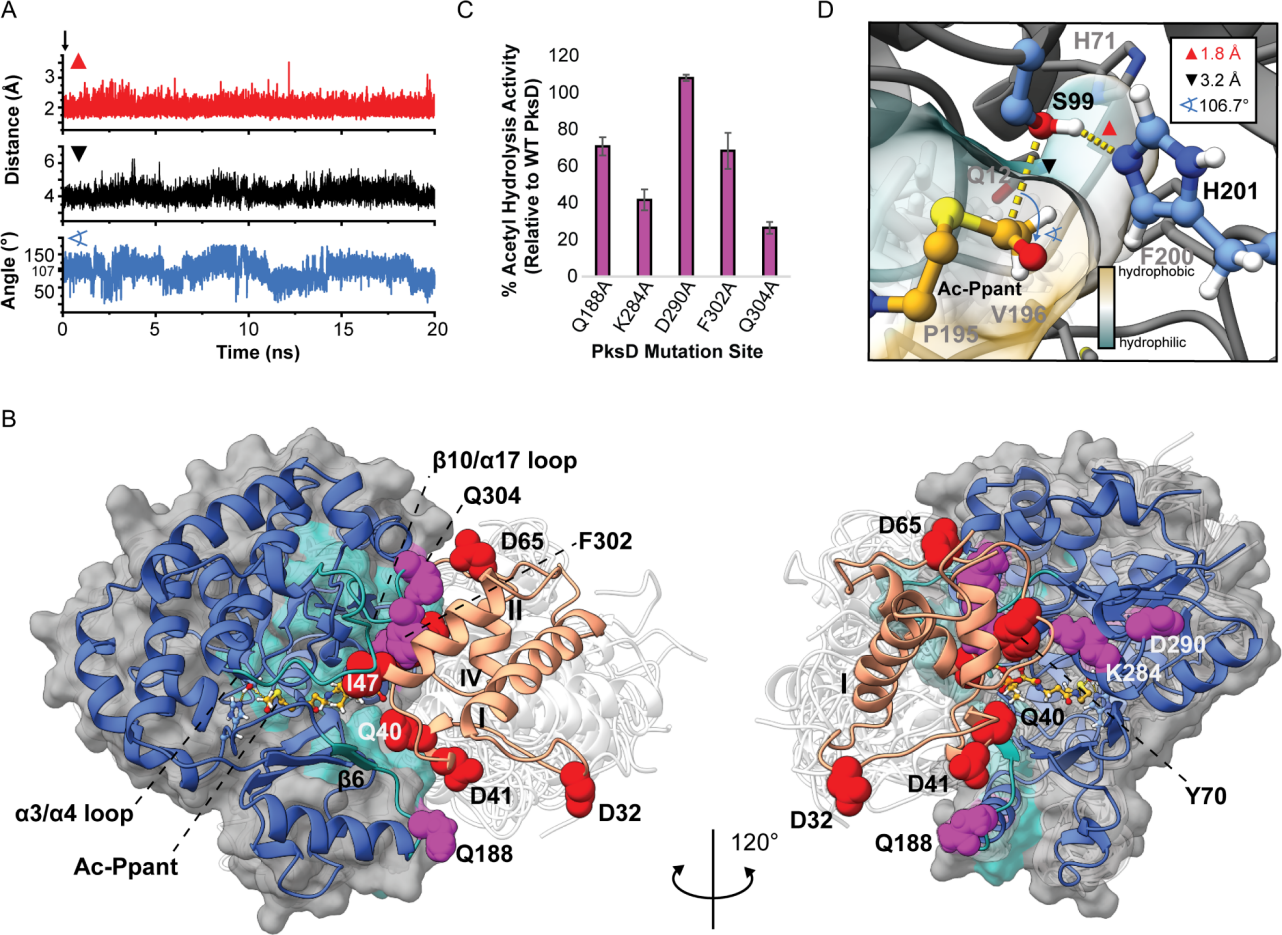
Representative cMD frame of the docked complex of PksD with the acetyl-PksJ ACP4 domain. (A) Distance (Ser99[Oγ] → His201[Nε], red trace; Ser99[Oγ] → Ac[C1]-Ppant, black trace) and angle (Ser99[Oγ]-Ac[C1]-Ppant-Ac[O]-Ppant, blue trace) plots during a 20 ns cMD simulation of the PksD:acetyl-Ppant-PksJ ACP4 domain complex. (B) Overview of the PksD:acetyl-Ppant-PksJ ACP4 domain complex. The position of the illustrated frame along the trajectory is indicated with an arrow in panel A. PksD is rendered as a blue cartoon and light gray semitransparent surface and the PksJ ACP4 domain as a salmon cartoon. Regions of PksD that interact with the PksJ ACP4 domain during the course of the simulation are highlighted in teal. PksJ ACP4 domain surface residues identified by alanine scanning mutagenesis as being important for productive binding to PksD are shown as red spheres. Residues mutated on the PksD surface are shown as purple spheres, and acetyl-Ppant is shown as yellow ball-and-sticks. White semitransparent cartoons depict the range of conformations sampled by PksD and the PksJ ACP4 domain in a subsequent 576 ns aMD simulation. (C) Profile of acetyl group hydrolysis toward the PksJ ACP4 domain for surface mutants of PksD, expressed as a percentage of activity relative to WT PksD. (D) Magnified view of the substrate binding pocket. Semitransparent surfaces along the pocket are colored according to hydrophobicity (yellow for hydrophobic, cyan for hydrophilic). Semitransparent sticks illustrate the range of conformations explored by the acetyl-Ppant moiety during the 576 ns aMD simulation.

As discussed above, our alanine scanning mutagenesis and carbene footprinting experiments yielded near-residue level information about the AH:ACP domain interaction interface. Distance restraints from these data sets were applied to docking and MD simulations starting from our crystal structure of PksD and an AlphaFold model of the PksJ ACP4 domain.^42,43^ The *apo*-PksJ ACP4 domain was manually docked onto PksD using virtual reality in ChimeraX;^53^ acetyl-Ppant was then attached to Ser46 of the PksJ ACP4 domain and fed into the active site of PksD. The resulting complex was then energy-minimized and subjected to 50 ns of cMD followed by 576 ns of aMD to explore conformational changes. We found that the ACP domain rotates with respect to PksD and exhibits a degree of conformational plasticity (especially with respect to helices II and III; Figure S23). We also monitored geometries relevant to acyl transfer to Ser99 (i.e., steps I–II in Figure 3F) and from three aMD frames approaching attack we initiated further 20 ns cMD simulations. In all three simulations, multiple frames matched the criteria for acyl transfer (Figure 8, Figure S24 and Video S5).

In a representative frame (Figure 8), the ACP domain binds to PksD in an orientation nearly identical to that observed for the crystallized DisD AT:DisA ACP1 domain complex (PDB: 5ZK4) (Figure S25).^26^ However, the PksJ ACP4 domain is translated forward relative to the DisA ACP1 domain such that helix II nestles between loop β10/α17 (on the C-terminal extension of PksD) and the lengthy loop α3/α4 (on the front face of PksD)—two features absent from the DisD AT domain (Figure S25). This forward displacement also permits the compact helix I to associate with strand β6 and neighboring loops of PksD. Notably, AlphaFold Multimer,^54^ which became available during the preparation of this manuscript, predicts a similar position for the ACP domain as in our MD simulations, but rotated ∼180° around the axis of the PksD substrate channel (Figure S26A). Interestingly, AlphaFold Multimer predicts an analogous solution for the DisD AT:DisA ACP1 domain complex, with the ACP domain also rotated around the substrate channel when compared to the covalently tethered crystal structure (Figure S26B). Furthermore, the prediction of all ACP domain complexes from the bacillaene *trans*-AT PKS with PksD produced four different binding modes. Here, most solutions adopted the rotated orientation as predicted for the PksD:PksJ ACP4 domain and DisD AT:DisA ACP1 domain complexes; however, a subset agreed with the orientation from our docked model and the covalently tethered crystal structure (Figure S26C). It is worth noting that catalytic geometries for acyl hydrolysis could be obtained starting from the rotated complex, suggesting there may be more than one binding mode that permits catalysis (Figure S27).

To further interrogate the contribution of PksD to the interface, five residues positioned near the ACP domain binding site in our docked model were mutated to Ala (Gln188, Lys284, Asp290, Phe302, and Gln304), and tested for their effect on acetyl-ACP domain hydrolysis (Figure 8B,C). Large reductions in activity were observed for K284A and Q304A, both of which are intimately involved in the ACP domain interface. The Q188A and F302A mutations showed a moderate decrease in activity. We propose that Gln188 is involved in an encounter complex, perhaps interacting with Gln40 or Asp41 of the ACP domain during the initial association. The F302A mutation likely reduces some hydrophobic packing with helix II without a major disruption to the interface. Importantly, D290A had no effect on hydrolytic activity and is not expected to engage in any interactions with the ACP domain in our proposed complex (Figures 8B,C and S28). It should be noted that the effects of these mutations do not unambiguously discount the AlphaFold prediction for the complex, which could be a valid binding mode for the interface.

Epitope-forming residues on the PksJ ACP4 domain that were identified by alanine scanning mutagenesis (Figures 7A and S18) either sit directly at the AH interface (e.g., Gln40, Asp41 and Ile47) or engage in stabilizing interactions during the course of our MD simulations (e.g., Asp32, Asp65 and Tyr70) (Figure 8B). Moreover, helix α9 becomes more solvent-exposed due to lateral movement of 3_10_ helix α8 and the adjacent loop containing Asn159 and Ser160 (Figure S29). In agreement with our carbene footprinting data, helix α9 (residues 180–182) shows unmasking upon ACP domain binding (Figures 7C and S22).

A representative frame (Figure 8D) illustrates the spatial limitations of the substrate binding pocket in fitting long (>6 carbons) or branched acyl chains, although the pocket does possess some dynamic qualities (Figures 8D and S30). The channel features both hydrophobic and hydrophilic faces, which likely facilitates proper orientation of the acetyl-Ppant moiety for acyl transfer (Figure S30C,D). We also carried out MD simulations on a docked complex between PksD and the malonyl-PksJ ACP4 domain to investigate how the AH behaves toward this nonpreferred substrate. During the 280 ns aMD simulation, no single frame satisfies the required geometry (Figure S31). The malonyl group appears to be unable to acylate Ser99 due to transient H-bonds between the carboxylate group and a range of polar residues near the active site, in addition to steric factors in the active site.

## Conclusions

In conclusion, we performed the first structure–function analysis of an AH, which rescues stalled *trans*-AT PKSs by hydrolyzing short acyl chains aberrantly carried by ACP domains. Our crystal structure and biochemical assays allowed us to propose mechanisms for the selection and hydrolysis of short acyl (e.g., acetyl) chains carried by ACP domains but not by CoA. Moreover, our interrogation of the AH:ACP domain interface via scanning alanine mutagenesis coupled with LC-MS assays, carbene footprinting and MD simulations revealed a binding mode similar to that observed for an AT:ACP domain pair with small but significant deviations. It is also worth highlighting that hydrolytic activity was observed toward thioesters appended to carrier protein domains from *cis*-AT PKS and NRPS pathways, although at a significantly reduced degree. In these pathways, housekeeping TE_II_s substitute for AHs and are thus likely to cleave substrates from such carrier protein subtypes more efficiently.

TE_II_s appear to be more versatile enzymes; able to carry out transacylation reactions to downstream carrier protein domains in addition to hydrolysis.^55,56^ In contrast, AHs function solely as hydrolases, with KS domains catalyzing required transacylation events in the pathway, often using a KS^0^.^49^ Due to their shared fold and high sequence similarity, AHs are likely the result of functional repurposing of *trans*-acting ATs. Why TE_II_s from *cis*-AT PKSs have not been recruited to fulfill this same role (with the notable exception of an AT-TE_II_ didomain in the lactimidomycin pathway (Figure 1C)) is unclear. One plausible explanation is that TE_II_s fail to interact readily with ACP domains in *trans*-AT PKSs, meaning numerous mutations would need to be acquired for functional interaction. From an evolutionary perspective, this may present a significant hurdle that favors catalytic repurposing of an enzyme that already interacts with ACP domains in the required manner. Furthermore, it is well established that TEs possess a dynamic lid structure, which adopts “open” and “closed” conformations to allow access to the substrate binding channel.^10,57−60^ It is possible that the ACP:TE_II_ interaction plays a key role in controlling these conformations, thus making the TE_II_ able to interface effectively with only certain carrier protein domains. This may be particularly relevant for TE_II_s that catalyze transacylation reactions, as they seem to have specificity for a single carrier protein domain in the assembly line.^55,56^ Elucidating the molecular basis for productive TE_II_:ACP interactions is therefore the logical next step in developing a better understanding of the specificity and potential utility of these enzymes.

While AHs and ATs do not appear to undergo such drastic conformational changes, they must possess an epitope with sufficient plasticity to productively bind the diverse interaction surfaces presented by ACP domains in *trans*-AT PKSs. This diversity results from coevolution of amino acid residues at the binding interface of ACP domains and their partner catalytic domains, i.e., the upstream α/β-carbon modifying domains and downstream KS domain, consistent with the “mosaic” architecture of *trans*-AT PKSs.^61,62^ The required plasticity is likely achieved by presentation of a largely hydrophobic interface on the AHs and ATs to the ACP domains, as exemplified by PksC and PksD (Figure S32), with recognition relying on shape rather than specific electrostatic/H-bond interactions. This would permit multiple productive binding modes, as appears to be the case for the PksD:PksJ ACP4 complex we analyzed here (Figure S26) and was previously observed for the AT:ACP complex in the *E. coli* fatty acid synthase.^52^ It may also explain the low-level activity toward noncognate carrier protein domains we observed in this study (Figure 7B). However, the AT/ACP interface must also be tunable to enable specific interactions, as exemplified by loading of an ethylmalonyl-CoA extender unit onto a specific ACP domain of the kirromycin PKS by the KirCII *trans*-acting AT.^22^ In contrast, the module duplication events underpinning *cis*-AT PKS evolution have permitted far less sequence divergence within their ACP domains, implying that TE_II_s may employ more specific interactions to engage with their cognate carrier protein domains.

Taken together, our comprehensive study of AH structure, function, and mechanism provides important new perspectives on type I modular PKS enzymology, highlighting an intricate interplay between *cis* and *trans*-acting core catalytic components in *trans*-AT PKSs, which is required to ensure optimal biosynthetic efficiency and fidelity. This interplay merits careful consideration when designing future synthetic biology approaches to the creation of optimally productive engineered *trans*-AT PKS systems.^63^

## Data Availability

The structure factor amplitudes and atomic coordinates of PksD have been deposited in the RCSB Protein Data Bank under PDB codes 8AVZ (SeMet derivative) and 8AW0 (native protein).

## Supporting Information Available

Topology, trajectory files; biochemical, biophysical and computational methods; chemical synthesis; sequences, tables and accession numbers (PDF)
Videos S1–S5 for the MD simulations discussed in the manuscript can be found on Zenodo at https://doi.org/10.5281/zenodo.7657414(MP4) (MP4) (MP4) (MP4) (MP4)
